# CD276 is an important player in macrophage recruitment into the tumor and an upstream regulator for PAI-1

**DOI:** 10.1038/s41598-021-94360-9

**Published:** 2021-07-21

**Authors:** Sibel Durlanik, Katrin Fundel-Clemens, Coralie Viollet, Heinrich J. Huber, Martin Lenter, Kerstin Kitt, Stefan Pflanz

**Affiliations:** 1 Cancer Immunology and Immune Modulation, Boehringer Ingelheim Pharma GmbH & Co. KG, 88397 Biberach, Germany; 2 Global Computational Biology and Digital Sciences, Boehringer Ingelheim Pharma GmbH & Co. KG, 88397 Biberach, Germany; 3 Drug Discovery Sciences, Boehringer Ingelheim Pharma GmbH & Co. KG, 88397 Biberach, Germany

**Keywords:** Cancer, Cell biology, Immunology, Oncology

## Abstract

More than 70% of colorectal, prostate, ovarian, pancreatic and breast cancer specimens show expression of CD276 (B7–H3), a potential immune checkpoint family member. Several studies have shown that high CD276 expression in cancer cells correlates with a poor clinical prognosis. This has been associated with the presence of lower tumor infiltrating leukocytes. Among those, tumor-associated macrophages can comprise up to 50% of the tumor mass and are thought to support tumor growth through various mechanisms. However, a lack of information on CD276 function and interaction partner(s) impedes rigorous evaluation of CD276 as a therapeutic target in oncology. Therefore, we aimed to understand the relevance of CD276 in tumor-macrophage interaction by employing a 3D spheroid coculture system with human cells. Our data show a role for tumor-expressed CD276 on the macrophage recruitment into the tumor spheroid, and also in regulation of the extracellular matrix modulator PAI-1. Furthermore, our experiments focusing on macrophage-expressed CD276 suggest that the antibody-dependent CD276 engagement triggers predominantly inhibitory signaling networks in human macrophages.

## Introduction

CD276/B7-H3 is a type I transmembrane protein and, based on the similarity in protein sequence, is considered as an immune checkpoint member of the B7 family. In mouse, the extracellular portion of CD276 is composed of two immunoglobulin (IgV–IgC) domains, whereas in human it has four Ig domains of two pairs of IgV–IgC due to exon duplication^1^. Despite being discovered almost 2 decades ago, the interaction partner(s) remains unknown. The short cytoplasmic tail lacks any known adaptor protein motif^2^, leaving the downstream signaling of CD276 unresolved. Initially, the studies mainly focused on modulation of T cell responses by CD276, yet the reported functions as co-stimulatory and co-inhibitory molecule remain controversial^2–5^. In addition to its debated immune modulatory functions, CD276 gained considerable attention in the tumor field. CD276 is expressed on fibroblasts^6^, epithelial cells^7^, endothelial cells^8^ as well as stromal cells^9^, and CD276 overexpression has been detected on a vast variety of solid tumors such as colorectal, breast and lung cancer^10^. The large body of data indicates a strong correlation between CD276 overexpression in tumor and poor patient prognosis^10^. For example, in hepatocellular carcinoma, CD276 expression associated with tumor aggressiveness and recurrence rate^11^. Nuclear localization of CD276 in colorectal cancer, high CD276 expression in pancreatic cancer and on tumor-associated endothelial cell in ovarian carcinoma were reported to be a predictive parameter for reduced survival rate^12–14^. Its pro-tumorigenic functions so far were associated to tumor growth, glucose and fatty acid metabolism, cell migration, metastasis, drug and chemotherapy resistance^15–18^.

Multiple studies suggest a possible connection between poor clinical prognosis in patients with high CD276 expression on tumor cells and lower tumor infiltrated lymphocytes such as CD8^+^ T cells, which implies an immune-modulatory role of CD276^19–22^. Analysis of tissue sections obtained from colorectal cancer patients indicated a positive correlation between CD276 expression on tumor cells and CD68^+^ tumor associated macrophages (TAM) infiltration^20^. Similarly, in hepatocellular carcinoma, CD276 expression was shown to positively correlate with infiltrated TAM and the same study indicated a potential role of tumor-expressed CD276 in polarization of the human monocytic cell line, THP-1 cells, to an anti-inflammatory M2-like phenotype^23^. Recently, a new study challenged this claim by demonstrating that higher CD276 expression on tumor cells positively correlated with increased number of CD8^+^ T cell and plasmacytoid dendritic cells (pDCs) in patients suffering from non-small cell lung cancer (NSCLC)^24^. Clearly, further studies are necessary to understand the connection between CD276 expression on tumor and tumor-infiltrating lymphocytes.

However, to date there is no systematic study available investigating a possible role of CD276 on tumor in macrophage infiltration. Therefore, in this study, we aimed to understand whether CD276 modulates macrophage recruitment in a 3D tumor spheroid with human macrophage coculture system. Our data suggest that tumor-expressed CD276 contributes to macrophage recruitment in spheroid, possibly by influencing extracellular matrix (ECM) remodeling. In the absence of CD276 on tumor cells, plasminogen activator inhibitor-1 (PAI-1) and urokinase plasminogen activator (uPA)—two recognized players in ECM remodeling—are affected, indicating that CD276 could be an upstream regulator for PAI-1 in tumor. In addition to tumor cells, our data also provide insights in CD276 function on macrophages. CD276 is upregulated during monocyte differentiation to macrophages, and our data show that the intensity of CD276 expression depends on the cytokine milieu. Cross-linking of CD276 on macrophages with a specific antibody indicated a potential *cis*-partnership leading to phosphorylation of ITIM bearing LAIR-1, PECAM-1, Siglec-3, ILT2, ILT4 and ILT5 accompanied with kinase phosphorylation such as CREB, HSP27, ERK1/2, RSK1/2/3, c-Jun and STAT3. In conclusion, our data suggest that tumor-expressed CD276 is involved in regulating the recruitment of tumor-associated macrophages and provide insights, for the first time, in signaling mediated by CD276 in macrophages.

## Material and methods

### Cell culture and monocyte differentiation to macrophages

HCT116 colon cancer cells (ATCC, CCL-247) were propagated and maintained in McCoy’s 5A (Modified) medium (16600082, Gibco) with 10% FCS supplement.

Monocytes were isolated from frozen PBMCs obtained from either fresh blood donation or Leukopak concentrates. All studies on human donor blood were performed in accordance with the guidelines and regulations of German legislation and the experimental protocol was approved by the ethical committee of the Landesärztekammer Baden-Württemberg (Germany). Anonymized blood donors were healthy volunteers who provided written informed consent. PBMCs were isolated via density gradient centrifugation using Ficoll-Paque (17-1440-03, GE Healthcare) and stored in − 160 °C until further use. CD14^+^CD16^−^ Monocytes from frozen PBMC were magnetically isolated by using RoboSep according to the company’s protocol (EasySep monocyte isolation kit 19359, STEMCELL technologies). Isolated cells were seeded in 6-well Nunc plates at 1 × 10^6^ cells/ml with UpCell surface (174901, Thermo Fisher) in serum-free X-VIVO-10 media (BEBP02-055Q, Lonza) and differentiated to macrophages by incubation for 7 days with 100 ng/ml M-CSF (130-096-489, Miltenyi Biotec) or GM-CSF (130-093-866, Miltenyi Biotec).

### Crispr-Cas9 gene editing for HCT116 cells and macrophages

Neon Transfection System (MPK1096, Invitrogen) was utilized to deliver the Cas9 (A36499, Invitrogen)—ribonuclear proteins (RNP) complex including guiding RNA against CD276 (A35533-CRISPR806748_SGM, Invitrogen) or scrambled gRNA (A35526, Invitrogen) as control into the cell according to the company’s protocol with following modification.

HCT116 cells were treated with accutase (A6964, Sigma Aldrich) to detach and washed with PBS. For each transfection, 5 × 10^4^ cells were electroporated with RNP complex (240 ng gRNA + 1.25 µg Cas9) in 10 µl reaction buffer with following setting on Neon: 1530 V, 20 ms, 1 pulse. After electroporation, cells were seeded in 24-well culture plate (142475, Thermo Fisher) in McCoy’s 5A (modified) medium with 10% FCS supplement and cultured for 4 days. CD276 expression on the cell surface was analyzed by flow cytometry.

Monocyte-derived macrophages (MDM) at day 7 were collected, washed with PBS and prepared for electroporation. For each transfection, 2 × 10^5^ cells were electroporated with the RNP complex (240 ng gRNA + 1.25 µg Cas9) in 10 µl reaction buffer with following setting on Neon: 1650 V, 10 ms, 3 pulse. After electroporation, cells were rested in X-VIVO-10 medium supplemented with 100 ng/ml M-CSF or GM-CSF for 7 days. CD276 expression was analyzed on day 7 by flow cytometry.

### RNA isolation and quality control

HCT116^*WT*^ and HCT116^*CD276KO*^ cells were cultured and maintained in different culture flasks for further propagation. Five biological replicates were used per group for a total of 10 samples. 1 × 10^6^ cells per sample were collected, centrifuged, washed two times with PBS. After removal of supernatant completely, cell pellets were immediately frozen at − 80 °C until further use. Total RNA extraction for Next-Generation Sequencing from cell pellets lysed in RLT plus buffer were performed by using the AllPrep DNA/RNA Mini Kit (80224, Qiagen) according to the company’s protocol. An on-column DNA digestion was performed, and the final elution volume was 30 µL.

Total RNA was quantitatively and qualitatively assessed using the fluorescence-based RNA Broad Range Qubit Kit (Thermo Fisher) and the Standard Sensitivity RNA Analysis DNF-471 Kit on a 48-channel Fragment Analyzer (Agilent), respectively. Concentrations averaged at 30 ng/µL while RIN were all > 8.5.

### Whole transcriptome sequencing

10 HCT116-derived RNA samples were normalized and 200 ng of input was used for library construction using the NEBNext Ultra II Directional RNA Library Prep Kit for Illumina (E7760), together with the NEBNext Poly(A) mRNA Magnetic Isolation Module (E7490) upstream and the NEXNext Multiplex Oligos for Illumina (E7600) downstream (all New England Biolabs). Sequencing libraries were quantified, normalized, pooled and spiked in with PhiX Control v3 (Illumina). The library pool was subsequently clustered with the HiSeq 3000/4000 SR Cluster Kit on a cBot; and sequenced on a HiSeq 4000 Sequencing System (Illumina) with dual index, single read at 50 bp length (Read parameters: Rd1: 51 Rd2: 8, Rd3: 8), reaching an average depth of 40 million Pass-Filter reads per sample (11.1% CV).

### RNA-seq data analysis

Demultiplexing was performed using bcl2fastq v2.20.0.422 from Illumina. Sequencing reads from the RNA-seq experiment were processed with a pipeline building upon the implementation of the ENCODE “Long RNA-seq” pipeline^25^: filtered reads were mapped against the Homo sapiens (human) genome hg38/GRCh38 (primary assembly, excluding alternate contigs) using the STAR (v2.5.2b) aligner^26^ allowing for soft clipping of adapter sequences. For quantification, transcript annotation files from Ensembl (v86) corresponding to GENCODE 25 were used. Gene expression levels were quantified with the above annotations, using RSEM (v1.3.0)^27^ and featureCounts (v1.5.1)^28^. Quality controls were implemented using FastQC (v0.11.5)^29^, picardmetrics (v0.2.4)^30^ and dupRadar (v1.0.0)^31^ at the respective steps. Finally, differential expression analysis was performed on the human mapped counts derived from featureCount using limma/voom^32,33^. Processed data can be accessed in the NCBI GEO database under the accession code (GSE165610).

### Spheroid formation and macrophage coculture

4000 cells from HCT116^*WT*^ or HCT116^*CD276KO*^ were seeded in 96-well (Ultra-Low Attachment, ULA) plates (7007, Corning) in McCoy’s 5A (Modified) medium and cultured for 5 days to form spheroid. On day 5, culture medium was replaced with serum-free X-VIVO-10 medium. In parallel, MDMs were collected, washed with PBS and resuspended in X-VIVO-10 medium. 1 × 10^4^ MDMs were distributed per spheroid and cocultured for additional 4 days. At the end of coculture, supernatants were collected and stored at − 20 °C until further use. To quantify the viable cells per spheroids, 5–10 spheroids were pooled into one sample for each group and before dissociating the spheroids, a defined number of negative compensation flow cytometry beads (51-90-900129, BD Bioscience) were added. Afterwards, spheroids were dissociated, and single cell suspensions were processed for surface and intracellular staining. Upon analysis by flow cytometry, ratio of recorded bead count to the initial bead amount was used to back calculate viable cells. For the experiments with uPA, recombinant uPA protein (ab167714, Abcam)—2 ng/ml or 20 ng/ml—was added to the spheroid-MDM coculture on day 5. For the experiments with αCD276 antibody treatment, only HCT116^*WT*^ spheroids were used and cocultured with MDMs. The antibody treatment experiment was performed in the presence of 20 µg/ml Fc Block (564765, BD Bioscience), and cells received either 20 µg/ml αCD276 antibody (ab209895, Abcam) or 20 µg/ml monoclonal rabbit IgG isotype (ab199376, Abcam) for 4 days.

### Flow cytometry

Single cell suspension was washed with 0.2% BSA-PBS and directly incubated with Pacific Orange (P30253, Thermo Fisher) for 10 min on ice to exclude dead cells for the follow-up data analysis. After live/dead staining, surface staining was carried out with the listed antibodies in the presence of αhFcγ‐receptor (130-059-901, Miltenyi Biotec) in 0.2% BSA‐PBS for 10 min on ice. After washing, to proceed with intracellular CD68 staining, cells were fixed and permeabilized with FACS‐Lysing (349292, BD Bioscience) and FACS‐Perm2 Solution (340973, BD Bioscience), respectively, according to the company’s protocol. Cells were analyzed using the flow cytometer FortessaX20 (BD Bioscience).

Following antibodies with indicated dilutions were used: BD Bioscience; CD14 (564054; 1:100), CD16 (560717; 1:50), CD163 (562643; 1:50), CD206 (550889; 1:50), CD36 (561820; 1:20), CD86 (563412; 1:50), CD68 (564944; 1:200), CD209 (558263; 1:20), CD11b (557960; 1:100), CX3CR1 (565801: 1:50), CD83 (564441; 1:25), Siglec1 (742995; 1:50); HLA-DR (564516; 1:50). BioLegend; CD200R (329310; 1:100), CCR2 (357204; 1:50), CD11c (337220; 1:200). Miltenyi Biotec; CD300E (130-101-777; 1:50) and CD276 (130-095-525; 1:100). eBioscience; Marco (25-5447-42; 1:20).

For the samples in which unconjugated rabbit αhCD276 (ab209895, Abcam) was added to the culture, the flow cytometric analysis of CD276 surface expression was performed with mouse αhCD276 (130-095-525, Miltenyi Biotec).

### MDM stimulation

MDMs generated for 7 days were collected, washed with 0.2% BSA‐PBS, and resuspended in serum-free X-VIVO-10 medium with a cell density of 1 × 10^6^ cells/ml. MDMs were reseeded into 48-well or 24-well Nunc cell culture (UpCell) plates. After 2 h resting, following stimuli were added to the culture: 20 ng/ml LPS (tlrl-b5lps, InvivoGen) + 50 ng/ml IFNγ (571104, BioLegend); 10 ng/ml IFNα (11100-1, PBL); 20 ng/ml IL-4 (130-093-922, Miltenyi Biotec) + 20 ng/ml IL-13 (571104, BioLegend); 20 ng/ml IL-10 (571004, BioLegend). 24 h after stimulation, supernatants were collected and stored at − 20 °C until further use.

### Soluble protein analysis

To quantify the protein amount in supernatants, following ELISA kits and arrays were used according to the company’s protocol: PAI-1 ELISA (BMS2033, Thermo Fischer); uPA ELISA (DUPA00, R&D System); Proteome Profiler Human XL Cytokine Array (ARY022B, R&D System).

### WST-1 assay

Spheroids from HCT116^*WT*^ and HCT116^*CD276KO*^ cells were formed as described above and on day 5, culture medium was exchanged to serum-free X-VIVO-10 medium. From day 6 on, metabolic activity was assessed using the WST-1 assay kit according to the company’s protocol (Roche, 11644807001). Media was aspirated and 150 μl of 10% WST-1 solution was added to each well and cells were incubated for 3 h. To measure the absorbance at wavelength 450 nm, 100 µl from the supernatant was transferred into a 96-well F-bottom plate and immediately was recorded using microplate reader (EnVision, PerkinElmer).

### Phospho-immunoreceptor and phospho-kinase arrays

MDMs were generated as described above. On day 7, cells were collected, washed with 0.2%BSA-PBS and reseeded in 24-well UpCell plates with a density of 1–1.5 × 10^6^ in 500–750 µl X-VIVO-10 medium. After 3 h resting, cells were treated with 20 µg/ml Fc Block (564765, BD Bioscience) for 10 min at 37 °C. Afterwards, cells were stimulated with either 20 µg/ml αCD276 antibody (ab209895, Abcam) or 20 µg/ml monoclonal rabbit IgG isotype (ab199376, Abcam). For Phospho-Immunoreceptor array (ARY004, R&D System), antibody treatment was for 10 min at 37 °C and 30 min for Phospho-Kinase Array (ARY003c, R&D System). After incubation, cells were immediately transferred onto ice, collected and washed 2 times with PBS. Cell lysate preparation was carried out according to the company’s protocol. To inhibit the proteinase activities, lysis buffers for all samples were supplemented with 1% Halt Protease Inhibitor Cocktail (EDTA free) (87785, Thermo Fisher). Cell lysates were stored at − 80 °C until further use. Protein quantification was performed with Pierce BCA Protein Assay (23225, Thermo Fisher) and measurement was carried out with microplate reader (EnVision, PerkinElmer). Equal amount of cell lysates (between 200 and 250 µg) was used per assay, and membranes were developed with ImageQuant LAS 4000 (GE Healthcare).

### Statistical analyses

GraphPad Prism was used to perform statistical analyses. Paired student *t* test was used to calculate *p* values. *p ≤ 0.05; **p ≤ 0.01; ***p ≤ 0.001.

## Results

### CD276 expression on the HCT116 colorectal cell line is dispensable for tumor spheroid formation

**Figure 1:**
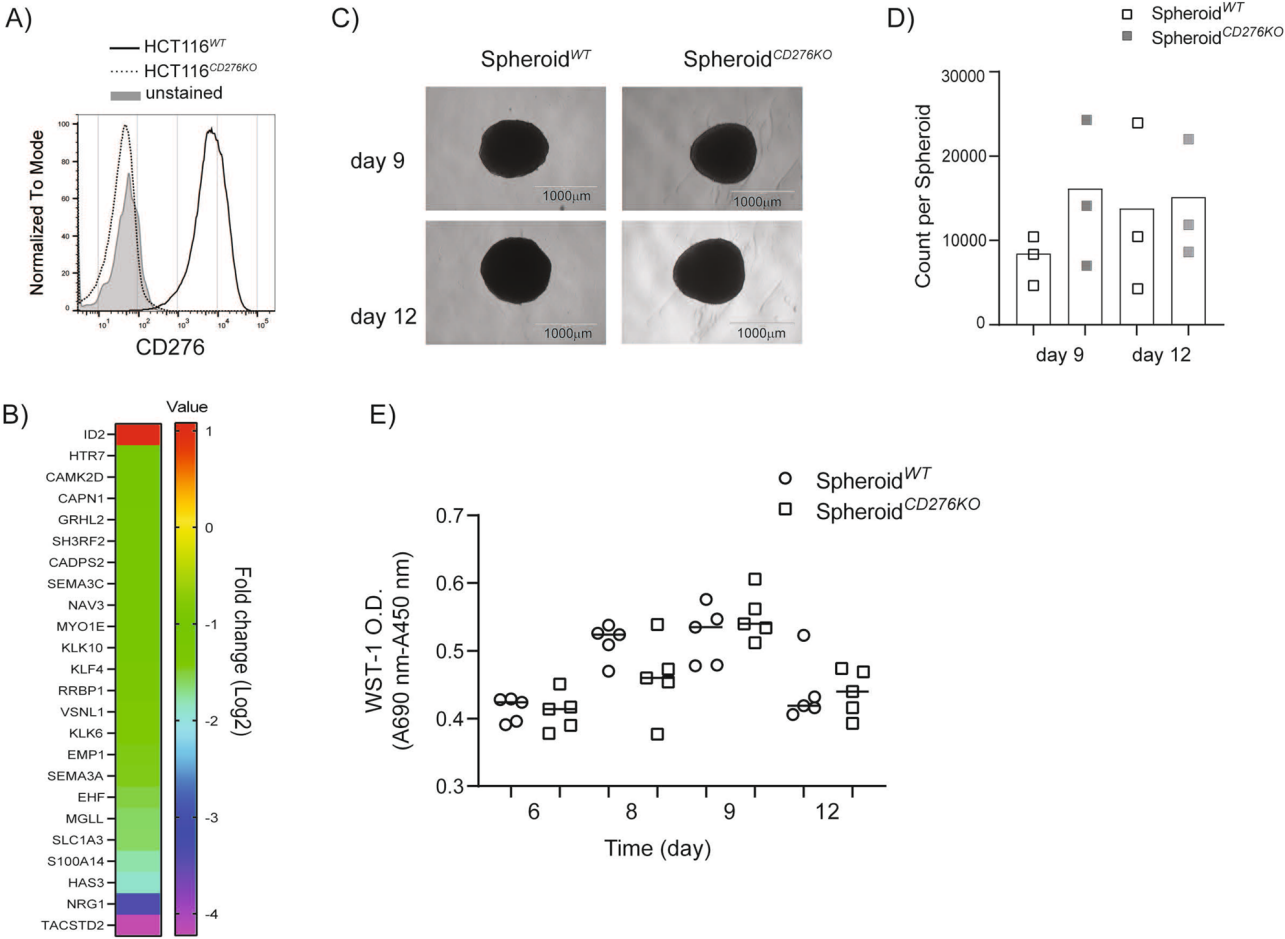
HCT116^*CD276KO*^ cells form tumor spheroids similar to HCT116^WT^ cells. (**A**) CD276 expression on HCT116^WT^ and HCT116^*CD276KO*^ cells was analyzed by flow cytometry after Crispr-Cas9 gene editing. (**B)** The heat-map shows the differentially regulated genes related to cell adhesion and migration in HCT116^*CD276KO*^ cells compared to HCT116^*WT*^ cells according to global transcriptome analysis. The bar shows the fold change (log2) of the ratio from tpm values (transcripts per kilobase million) of genes in HCT116^*CD276KO*^ cells to genes in HCT116^*CD276WT*^ cells. (**C)** Tumor spheroids formed from HCT116^*WT*^ and HCT116^*CD276KO*^ cells were monitored up to 12 days for shape. Representative microscope images are from 9-day and 12-day culture. (**D)** Quantification of viable tumor cells per spheroid was assessed by flow cytometry. 5–10 spheroids were pooled into a single sample for each group and before dissociation a defined number of negative compensation flow cytometry beads were added. Ratio of recorded bead count to the initial bead amount was used to back calculate viable tumor cells. The graph summarizes data (Mean + SD) from three independent experiments. (**E)** WST-1 viability assay treatment of spheroids over time. Each dot is one spheroid.

HCT116 cells form spheroids in 5 days when cultured in a cell culture plate with ultra-low attachment (ULA) feature without additional support such as matrigel. HCT116 spheroids are rigid enough for transfer without disruption and permissive enough for monocytes and macrophages to infiltrate. HCT116 cells naturally express CD276, therefore we generated HCT116^*CD276KO*^ cells by employing Crispr-Cas9 gene editing technology. The loss of CD276 on the cell surface was monitored and HCT116^*CD276KO*^ cells were sorted by flow cytometry to generate single cell clones (Fig. 1A). All the experiments were performed with the same single cell clone. Our next step, global transcriptome analysis revealed that 55 genes were differentially regulated in HCT116^*CD276KO*^ cells compared to HCT116^*WT*^ cells. In silico analysis of potential off-targets of the CD276 specific gRNA performed with CrisprOR software^34^ yielded no possible site for up to two-mismatch and 66 genes if four-mismatch were allowed. However, none of these overlapped with the gene set found after CD276 deletion (Supplementary Table S1). Despite being a short list of genes, when analyzed with IPA (Ingenuity pathway analysis), about 25 genes were associated with cell motility and adhesion (Fig. 1B). Although the pathway analysis indicated a potential role for CD276 in extracellular matrix (ECM), HCT116^*CD276KO*^ cells as well as HCT116^*WT*^ cells formed comparable round, intact spheroids from 5 to 12 days with no visible distortion (Fig. 1C; Supplementary Fig. 1). We assessed the cell viability in spheroids by flow cytometry and WST-1 metabolic assay. Spheroids^*CD276KO*^ tended to have a higher percentage of viable cells although the difference was not statistically significant (Fig. 1D), but according to the WST-1 assay, cell viability in spheroids formed from HCT116^*CD276KO*^ and HCT116^*WT*^ cells was comparable as analyzed with unpaired student *t *test (Fig. 1E). Therefore, we concluded that lack of CD276 expression on tumor cells has no significant impact on HCT116 cell spheroid formation.

### Monocyte to macrophages differentiation induces CD276 upregulation

**Figure 2:**
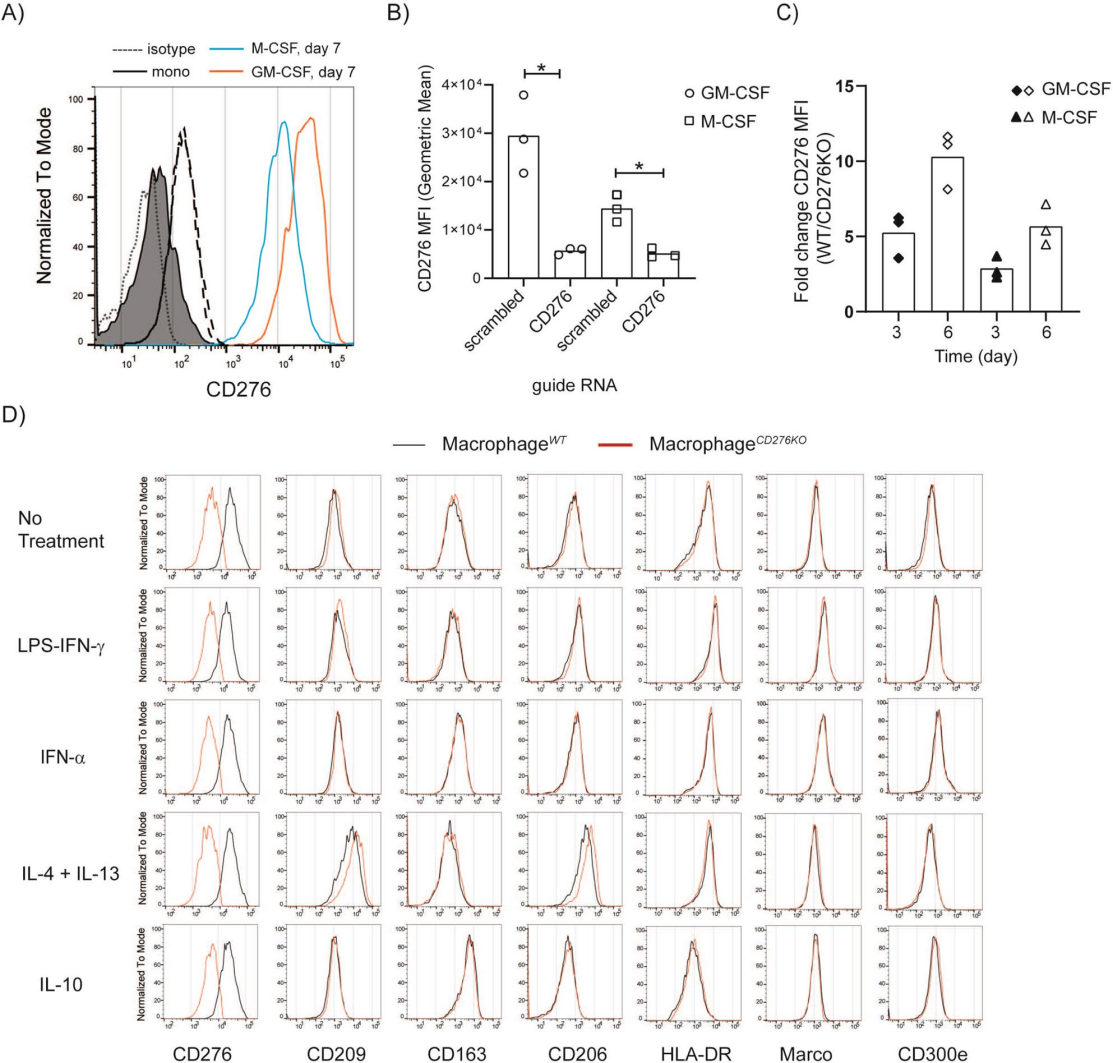
Monocyte to macrophage differentiation induces CD276 upregulation, yet lack of CD276 expression on macrophages shows no impact for further polarization. **(A)** CD276 expression was analyzed on freshly isolated monocytes (day 0) and monocyte-derived macrophages (MDMs/Macrophages) cultured for 7 days by flow cytometry. Black line, filled histogram: Monocytes; red line, open histogram: MDMs cultured in GM-CSF supplemented medium; blue line, open histogram: MDMs generated in M-CSF supplemented medium; dotted/dashed lines for day 0/7: isotype control. The histogram shows one representative donor out of four donors. (**B-D)** MDMs (M-CSF) were genetically edited with Crispr-Cas9 technique either with scrambled gRNA (Macrophage^*WT*^) as control or CD276-specific gRNA (Macrophage^*CD276KO*^). (**B)** The graph summarizes the mean fluorescence intensity (MFI, geometric mean) of CD276 on MDMs 3 days after gene editing. Statistical analysis was done with paired *t*-test * p < 0.05. (**C)** CD276 expression on MDMs was compared from day 3 to day 6 after gene editing by flow cytometry. The graph depicts the ratio of CD276 MFI on Macrophage^*WT*^ to Macrophage^*CD276KO*^ on indicated time points in GM-CSF or M-CSF supplemented condition. (**D)** 3 days after gene editing, MDMs (M-CSF) were further treated with indicated stimuli for 24 h and analyzed for indicated surface markers by flow cytometry. Red line: Macrophages^*CD276KO*^; black line: Macrophages^*WT*^. These histograms represent one representative donor out of three donors.

In addition to tumor cells, activated monocytes and macrophages were reported to express CD276^4,35^. We could confirm that freshly isolated human monocytes from frozen PBMCs lack CD276 but upregulate it while differentiating to macrophages in vitro when cultured in the presence of GM-CSF or M-CSF (Fig. 2A). In addition to these cytokines, coculturing with tumor spheroids (inside and outside of the spheroids) or exposing to tumor-conditioned medium (generated from tumor spheroids) for 7 days, and cell adhesion to the culture plate in the absence of GM-CSF or M-CSF also induced CD276 upregulation (Supplementary Figs. 2A, B). This indicates that multiple mechanism could induce CD276 expression on monocyte-derived macrophages.

### CD276 expression is dispensable for monocyte-derived macrophage (MDM) differentiation and further polarization

In spheroid-monocyte or -macrophage coculture, CD276 signaling comes not only from tumor but also from MDMs. During the crosstalk between tumor cells and MDMs, exchange of plasma membrane fragments—Trogocytosis^36^—might occur and tumor cells might acquire CD276 from MDMs. Hence, to avoid or minimize any possible surface CD276 exchange, we employed Crispr-Cas9 gene editing technology to monocytes and MDMs as well. 7 days after gene editing and culturing, maximum gene editing efficiency for CD276 in monocytes was about 55% (Supplementary Fig. 3A). This indicates that lack of CD276 in monocyte has no detectable impact on viability or differentiation to macrophages, since CD14, CD16, CD163, CD206 and CD209 expressions were also comparable in CD276^−^ and CD27^+^ T cells (Supplementary Fig. 3A). Crispr-Cas9 gene editing efficiency on monocytes for CD276 was not reliable or reproducible between donors. Therefore, for the following experiments, we focused only on MDMs. 3 days after gene editing, CD276 expression was significantly reduced on MDMs treated with CD276 gRNA compared to scrambled gRNA treated cells under M-CSF and GM-CSF condition and after 6 days the effect was stronger (Fig. 2B&C). CD276^*WT*^ MDMs and CD276^*low/KO*^ MDMs were further activated with LPS + IFNγ, IL-4 + IL-13, IFN-α and IL-10; and surface expression of CD209, CD163, CD206, HLA-DR, Marco, CD14, CD16, CD11b, CD11c, CD83, CD86, CD36 and CD300e were analyzed. As in line with literature data, the cytokine stimulation induced changes in the expression of the markers such as CD163, CD209, CD206, HLR-DR in M-CSF MDMs and in GM-CSF MDMs. However, as depicted in Fig. 2D and in Supplementary Fig. 3B&C, CD276^*low/KO*^ and CD276^*WT*^ MDMs displayed comparable expression pattern for the interrogated markers, confirming no gross impact of CD276 on macrophage polarization.

### CD276 expression on tumor spheroids is associated with increased macrophages infiltration

**Figure 3:**
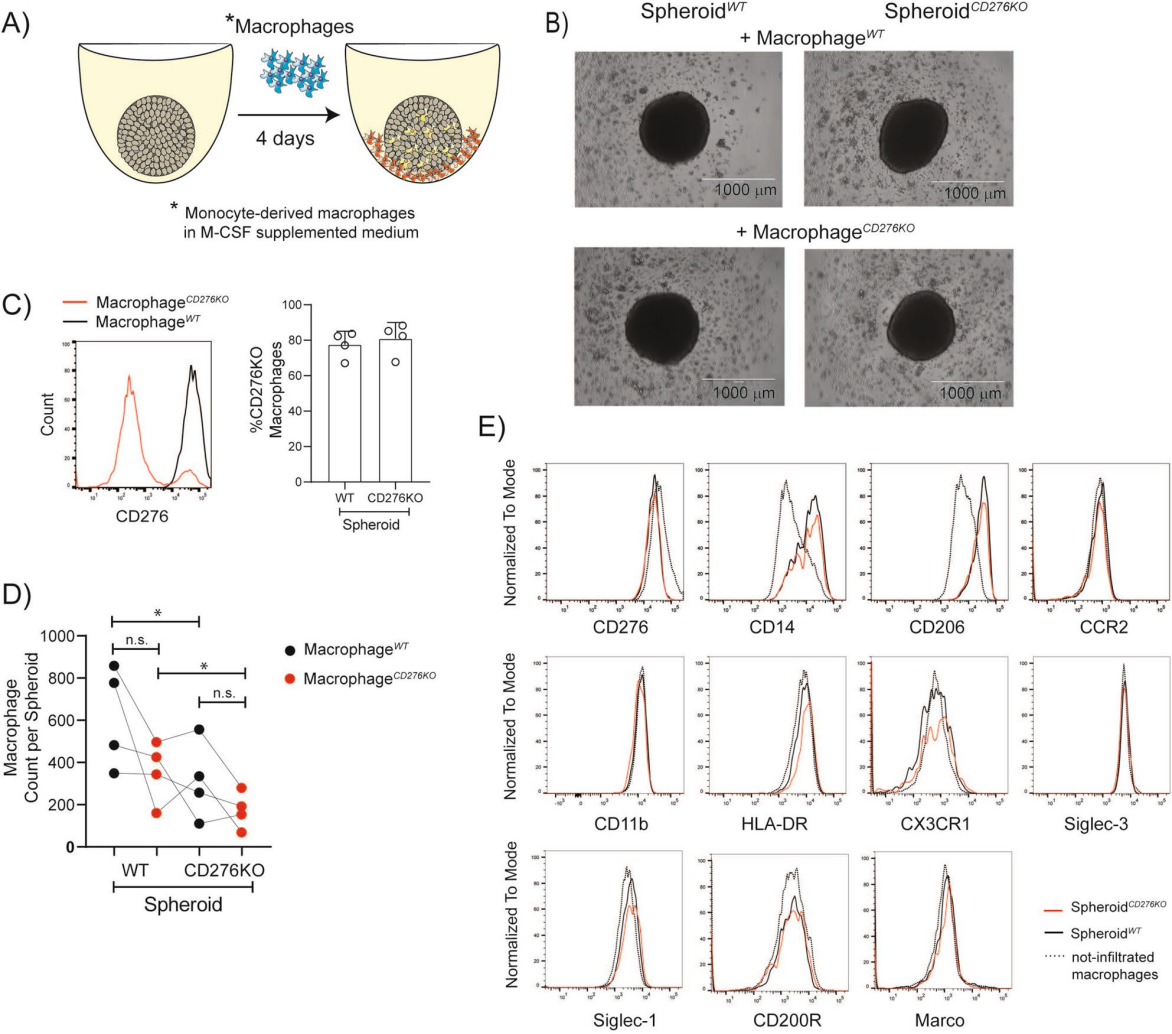
In the absence of CD276 on tumor cells, lower number of macrophages infiltrates into spheroids. Macrophages (MDMs in M-CSF supplemented medium) were genetically edited with Crispr-Cas9 protocol by CD276 specific gRNA (Macrophage^*CD276KO*^). Control cells were treated with scrambled gRNA (Macrophage^*WT*^). Afterwards, cells were rested for 7 days in culture and then cocultured with spheroids for another 4 days. CD11b^+^ MDMs inside the spheroids were analyzed by flow cytometry. (**A)** A schematic representation of experimental set-up for tumor spheroid culture with macrophages. 5 days after seeding HCT116^*WT*^ or HCT116^*CD276KO*^ cells, Macrophages^*WT*^ or Macrophages^*CD276KO*^ were added to the spheroids. 4 days after coculture, a fraction of macrophages infiltrated into the spheroid (yellow), while some macrophages (orange) remain attached in the well, outside of the spheroid. (**B)** Microscopy images show spheroid-macrophage cocultures for indicated conditions. (**C)** Histogram displays CD276 expression on Macrophage^*WT*^ (black line) and Macrophage^*CD276KO*^ (red line) in the spheroids. The graph summarizes the percentage of Macrophage^*CD276KO*^ among infiltrated macrophages Spheroid^*WT*^ and Spheroid^*CD276KO*^coculture from four different donors. (**D)** The count of infiltrated MDMs per spheroid is depicted in the graph. Briefly, 10 spheroids were pooled into a single sample for each group and before dissociation a defined number of negative compensation flow cytometry beads were added. Ratio of recorded bead count to the initial bead amount was used to back calculate MDMs. Macrophage^*WT*^ (black dots); Macrophage^*CD276KO*^ (red dots). Each dot represents one MDM donor. Statistical analysis was done with paired *t*-test; *p < 0.05. n.s. = not-significant **E)** Histograms display the surface expression of indicated markers on Macrophage^*WT*^ inside of Spheroid^*WT*^ (black line) versus Spheroid^*CD276KO*^ (red line) in comparison to not-infiltrated macrophages (dashed line) that were outside of the Spheroid^*WT*^. The data represent one donor from 4 donors MDMs (Macrophage^*WT*^).

To coculture with tumor spheroids, we used MDMs that were differentiated in M-CSF supplemented medium, since tumor-associated macrophages are linked to classically defined M2 or anti-inflammatory macrophages^37^ that have been found predominantly located in the intratumoral region and tumor invasive front^38^. Therefore, for the tumor spheroid cultures, the MDMs differentiated with M-CSF supplemented medium were viewed as the more relevant subset. As illustrated in Fig. 3A, once tumor spheroids were formed, MDMs were added and the coculture was maintained for 4 days. Unlike tumor cells, non-infiltrated macrophages attach on the ULA plates, which allows to collect spheroids selectively. The representative microscopic images of spheroids with macrophages coculture shows macrophage attachment at the bottom of the wells (Fig. 3B). Additionally, the detailed gating strategy for the macrophages in the spheroids can be found in Supplementary Fig. 4A. Consistent with previous publications, once macrophages infiltrated into the spheroid, they upregulated the expression of CD14, CD163 and CD206 compared to the macrophages outside of the spheroid (Supplementary Fig. 4B). There was also no difference in the shape or the size of these spheroids. As depicted in Fig. 3C, depending on the donor, the percentage of CD276^*KO*^ cells among macrophages was minimum 70% and this percentage was comparable in CD276^*WT*^ and CD276^*KO*^ spheroids. To quantify the infiltrated macrophages in the spheroids, we used the same bead-based technique as described above. Once cells were recorded in flow cytometry, the ratio of recorded beads to initial amount was used to back calculate the initial CD11b^+^ cells in the samples. As shown in Fig. 3D, 1.4 to 4.4-fold more macrophages were recruited into the Spheroid^*WT*^ compared to Spheroid^*CD276KO*^, independent of CD276 expression on macrophages. In our setting, there was no correlation between the count of infiltrated macrophages versus percentage of CD276-expressing macrophage. Although less macrophage infiltrated in the absence of CD276, the macrophages inside the CD276^*KO*^ and CD276^*WT*^ spheroids were comparable in the surface expression of CD14, CD206, CCR2, CD11b, HLA-DR, CX3CR1, Siglec-1, Siglec-3, CD200R and Marco (Fig. 3E and Supplementary Fig. 4C).

### In the absence of CD276, PAI-1 is increased while uPA is decreased

**Figure 4:**
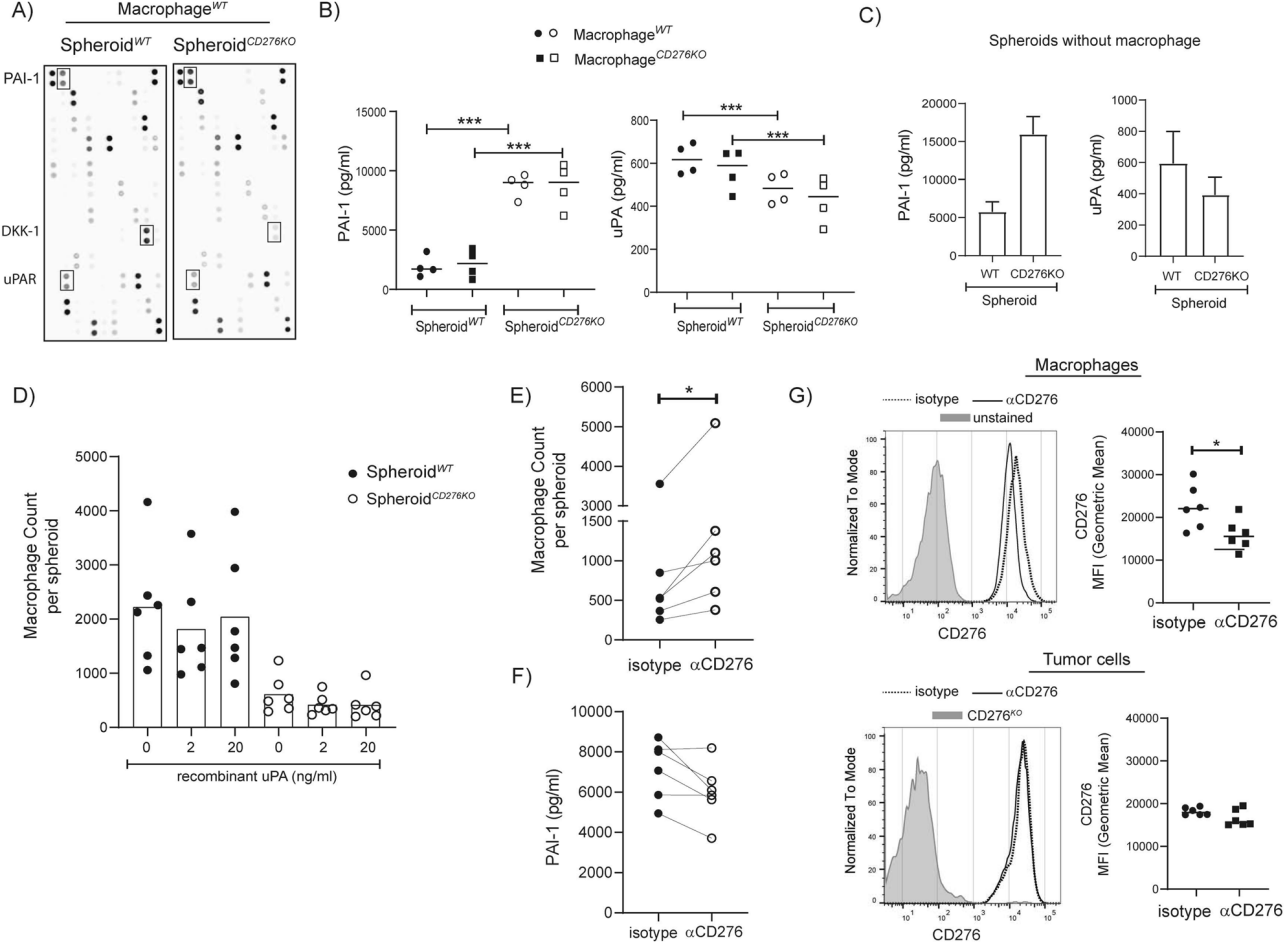
In the absence of CD276 on tumor, soluble PAI-1 is increased, while uPA is decreased. **(A&B)** Spheroids formed from HCT116^*WT*^ or HCT116^*CD276KO*^ were cocultured with Macrophages^*WT*^ or Macrophages^*CD276KO*^ (M-CSF) for 4 days and supernatants were collected. (**A)** Proteome Profiler Cytokine Arrays from equal volume of supernatants from Spheroid^*WT*^ or Spheroid^*CD276KO*^ cocultured with the same Macrophages^*WT*^ are presented here. (**B)** The graphs summarize the concentration of PAI-1 and uPA in the supernatants performed with relevant ELISA for indicated samples. (**C)** Spheroids were cultured without macrophages for the same period (5 days after initial seeding + 4 days after medium exchange). To collect enough supernatant, 5 wells from each group were pooled into one sample. PAI-1 and uPA concentrations were analyzed with ELISA. The data is from three independent experiments. (**D-F)** Spheroids formed from HCT116^*WT*^ or HCT116^*CD276KO*^ cells were cocultured with macrophages (7 days cultured in M-CSF supplemented medium, no gene editing) for 4 days. Quantifications for CD11b^+^ infiltrated macrophages were done by flow cytometry. 6–10 spheroids from each group were pooled into one sample, and before dissociating the spheroids, a defined number of beads (negative compensation beads for flow cytometry) was added. The ratio of recorded bead counts to initial bead amount was used to back calculate MDMs per spheroid. (**D)** Spheroids and macrophages were cultured in the absence or presence of recombinant uPA protein with indicated concentration. Six macrophage donors in total were tested. (**E–F)** Spheroid^*WT*^ cocultured with macrophages were treated with 20 µg/ml αCD276 antibody or isotype control. (**E)** Graph depicts the count of infiltrated macrophages per spheroid from six different donors. (**F)** The graph summarizes the concentration of PAI-1 in the supernatants from indicated samples (**G)** CD276 expression on macrophages and tumor cells treated with αCD276 antibody or isotype controls were analyzed. Histograms represent experiment with one macrophage donor and tumor cells in the same sample, and the graphs summarize the data from six donors and corresponding tumor cells. Dotted line indicates isotype treated controls, black line αCD276 antibody treated samples. Statistical analysis was done with paired *t*-test; **p < 0.01; ***p < 0.001.

Multiple cytokines and chemokines are described for monocyte and macrophage recruitment into the tumor such as CCL2 and CX3CL1^39^. To understand which soluble factor in our system may account for lower macrophage recruitment, we performed cytokine profile analysis on supernatants collected from spheroid-macrophage coculture. Three major soluble factors were affected in the absence of CD276 on tumor cells: Dickkopf-related protein 1 (DKK-1), PAI-1, urokinase-type plasminogen activator receptor (uPAR). Signal for PAI-1 was higher in supernatant from Spheroid^*CD276KO*^compared to Spheroid WT while the signals for DKK-1 and uPAR were lower (Fig. 4A). DKK-1 is a negative regulator for Wnt signaling, PAI-1 is an inhibitor for uPA that is the ligand for uPAR. PAI-1, uPA and uPAR are known players in ECM remodeling, cell adhesion and mobility^40^. Therefore, we decided to further quantify the PAI-1 and uPA concentration with ELISA. As in the cytokine array, significantly higher amount of PAI-1 was detected in the supernatant from Spheroid^*CD276KO*^ samples, while significant reduction in soluble uPA was observed in the same supernatant (Fig. 4B). The presence or absence of CD276 expression on macrophages in these samples exhibited no detectable impact on PAI-1 or uPA concentration. Hence, we quantified the PAI-1 and uPA amount in supernatants from spheroids without macrophages. As suspected, spheroids formed from HCT116^*CD276KO*^ cells produced threefold more PAI-1 and about 1.5-fold less uPA (Fig. 4C).

To investigate whether increased PAI-1 and as a result reduced uPA is the mechanism behind the reduced macrophage recruitment in the absence of CD276 on tumor cells, we added exogenous uPA to the spheroid-macrophage coculture (Fig. 4D). The uPA amount detected in spheroid cocultures were in the range of 300–800 pg/ml, therefore we added maximum 20 ng/ml recombinant uPA. As shown in Fig. 4D, addition of exogenous uPA could not restore the macrophage infiltration in the CD276^*KO*^ spheroids. According to PAI-1 quantification, exogenous uPA addition did not affect the PAI-1 concentration (Supplementary Fig. 6).

We tested a second method—αCD276-based immunotherapy approach—to modulate the macrophage infiltration via CD276. Targeting CD276 with an antibody is more clinically relevant, therefore we asked whether triggering CD276-mediated signaling would lead to opposite outcome. Spheroids formed from HCT116^*WT*^ cells were cocultured with macrophages (M-CSF) in the presence of αCD276 antibodies or isotype controls for 4 days. Based on quantification analysis done by flow cytometry, we detected increased macrophage infiltration upon αCD276 treatment (Fig. 4E). In parallel, we quantified the PAI-1 concentration in the supernatants, and in 4 out of 6 samples, up to 20–30% reduction in PAI-1 concentration was observed without statistically being significant when tested with student *t*-test (nonparametric) (Fig. 4F). Of note, αCD276 treatment displayed a selective effect on macrophages, but not on tumor cells. CD276 expression was selectively reduced on macrophages upon αCD276 antibody treatment, while tumor cells were not affected (Fig. 4G).

Confirming the role of CD276 in macrophage recruitment as well as in modulation of PAI-1 and uPA in cancer patients’ samples is essential to validate our data. Hence, we analyzed the colon adenocarcinoma cohort in The Cancer Genome Atlas (TCGA) and as shown in Supplementary Fig. 7, CD276 expression in tumor positively correlated with macrophage signature using the Xcell deconvolution method^25,41,42^.The coexpression analysis of CD276 and PAI-1 or uPA displays improved positive correlation in tumor samples compared to normal colon tissue that were obtained from GTEx project. Additionally, we analyzed publicly available single-cell RNAseq dataset generated from CRC patients’ samples^43^. In this data set, CD276 expression is mainly detected in TAM clusters and displays negative correlation with PAI-1 expression, whereas it is positively correlated to uPA expression, which supports our observation.

### Targeting CD276 on macrophages with a specific antibody induces a distinct immunoreceptor phosphorylation accompanied with kinase phosphorylation

**Figure 5:**
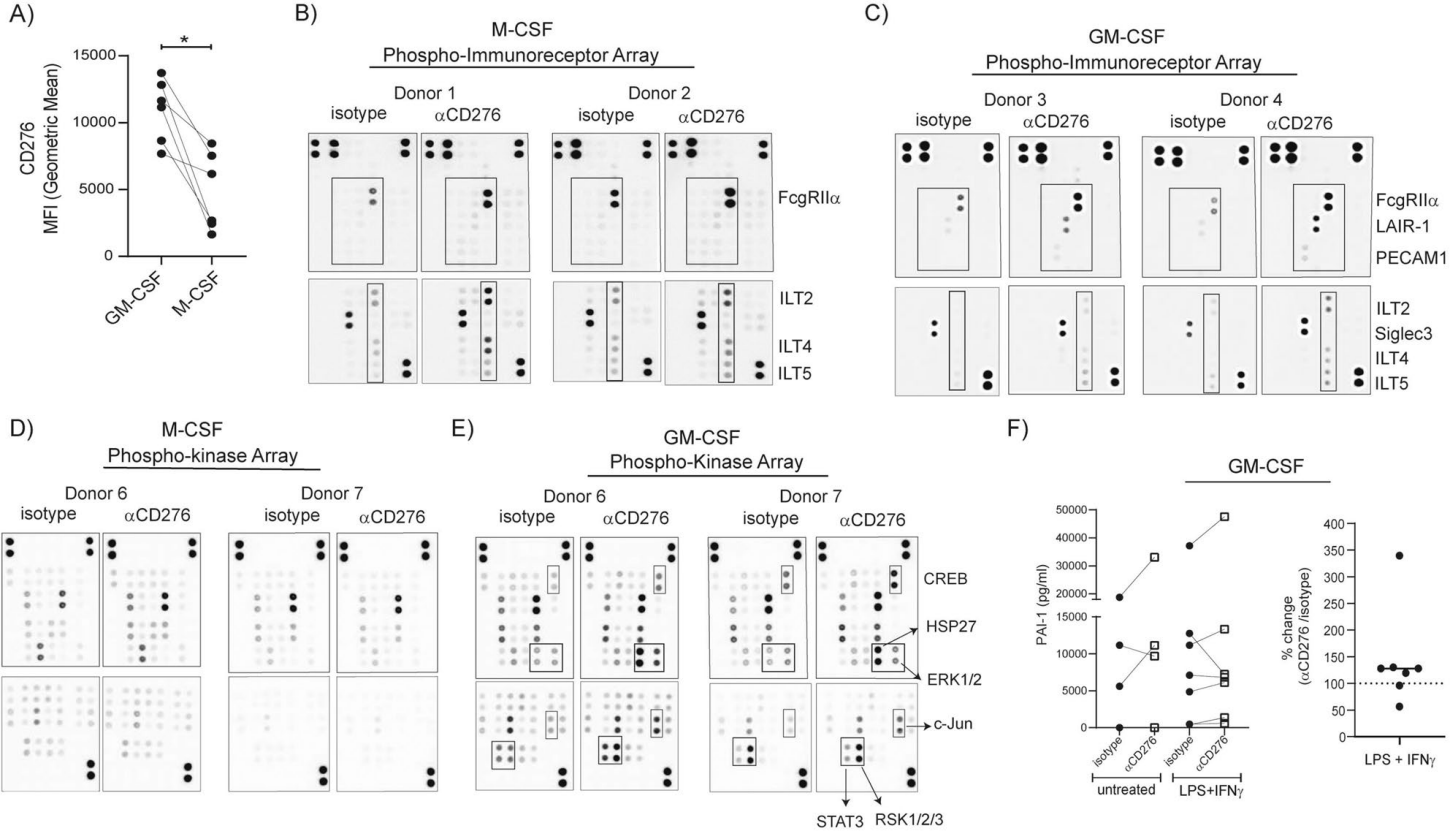
Targeting CD276 on macrophages with specific antibodies induces distinct immunoreceptor phosphorylation accompanied with kinase phosphorylation. (**A**) Monocytes were cultured either in GM-CSF or M-CSF supplemented medium for 7 days. CD276 expression on monocyte-derived macrophages (MDMs) was analyzed by flow cytometry and CD276 MFI values from six paired samples are summarized in the graph. (**B-E)** After 7 days culture, MDMs were reseeded for Phospho-Immunoreceptor and Phospho-kinase array. After 3 h resting, MDMs were treated with αCD276 antibody or isotype for 10 min for Phospho-immunoreceptor array and 30 min for Phospho-Kinase array. Cell lysates were processed for phosphorylation signatures. In total macrophages from 4–5 donors were tested. Two representative donors are shown for Phospho-Immunoreceptor arrays: (**B)** M-CSF MDMs and (**C)** GM-CSF MDMs. Two representative and matching donors are shown for Phospho-Kinase arrays: (**D)** M-CSF MDMs and (**E)** GM-CSF MDMs. (**F)** MDMs (GM-CSF) were left untreated or treated with LPS + IFNγ in the presence of αCD276 antibody or isotype control for 24 h. The graphs summarize the concentration of PAI-1 in the supernatants. Each dot represents one donor.

Selective effect of αCD276 antibody treatment on macrophages in spheroid coculture raised our interest for CD276-mediated signaling in macrophages. GM-CSF and M-CSF treatment induced CD276 upregulation, where GM-CSF induced significantly higher CD276 expression (Fig. 5A). Therefore, for the following analysis, we used both types of MDMs generated in M-SCF or GM-CSF supplemented medium. Despite having a short cytoplasmic tail with no known motif for any adaptor protein, CD276 was claimed to induce kinase phosphorylation^16^. If CD276 induces downstream signaling, this could indicate a cis-acting transmembrane protein partner. Immunoreceptors are among the main modulators for macrophages, therefore αCD276 treated MDMs were analyzed with Phospho-Immunoreceptor array (Fig. 5B&C). As shown from two representative donors, αCD276 treatment induced different immunoreceptor phosphorylation in M-CSF and GM-CSF generated MDMs. While ILT2, ILT4 and ILT5 were phosphorylated in M-CSF generated MDMs, LAIR-1, PECAM1 and Siglec-3 phosphorylation were dominating in GM-CSF generated MDMs. The further analysis for kinase phosphorylation upon αCD276 treatment demonstrated very distinctive pattern. Phosphorylation of CREB, HSP27, ERK1/2, c-Jun, STAT3 and RSK1/2/3 were observed in GM-CSF generated MDMs, almost no kinase phosphorylation in this setting was detected in M-CSF generated MDMs (Fig. 5D&E).

The detected immunoreceptors, LAIR-1, PECAM1, Siglec-3, ILT2, ILT4 and ILT5 possess ITIM inhibitory motif and perform inhibitory functions. To understand whether PAI-1 or uPA is the target of CD276 signaling in macrophages too, we activated MDMs in the presence of αCD276 antibody for 24 h with the following stimuli: LPS + IFNγ; IFNα; IL-4 + IL-13; or IL-10. We quantified PAI-1 and uPA in the supernatant from activated MDMs. MDM (M-SCF) produced detectable PAI-1 only under LPS + IFNγ condition with no clear impact from αCD276 antibody treatment (Supplementary Fig. 5). On the other hand, some MDMs (GM-CSF) donors produced PAI-1 already in unstimulated condition and often LPS + IFNγ stimulation increased the production (Fig. 5F). In MDMs (GM-CSF), αCD276 treatment tended to increase the PAI-1 production from 20 to 340% in four donors out of six donors (Fig. 5F).

## Discussion

Tumor-associated macrophages (TAM) compose up to 50% of tumor mass and are often supporters for tumor growth^44^. Heterogeneous composition of TAM subsets in the tumor microenvironment (TME) is becoming increasingly clear. In addition to promoting angiogenesis, tumor cell invasion and motility, TAM can suppress the anti-tumor T cell responses^45^. In this matter, CD276 as a member of the B7 family of immune checkpoint proteins is a good candidate, especially because the tumor express much higher CD276 compared to healthy tissue^10^. In addition to tumor cells, CD276 expression on macrophages was shown in non-small-cell lung cancer (NSCLC) patient samples^24^ as well as on TAM in in triple-negative breast cancer patient samples^46^. However, insufficient data about the CD276 function is an impediment to evaluate CD276 as a therapeutic target. According to correlation studies, patients with high CD276 expression are reported to have lower tumor infiltrating lymphocytes, without giving any insight about the underlying mechanism^10^. In this study, we employed 3D human tumor spheroids to recapitulate solid tumor structure and cocultured with human macrophages to monitor tumor-macrophage interactions, including macrophage infiltration into the tumor spheroid. Tumor spheroids can contain regions of proliferating, oxygenated, hypoxic and necrotic cells and thus are a good translational model for the situation in vivo^47^. Additionally, and as would be expected, 3D structure influences gene expression, cell motility and adhesion^47,48^. Therefore, using tumor spheroids allowed us to interrogate the correlation analysis results in an in vitro experimental setting with human cells. Our data suggest that CD276 on tumor cells is a significant contributor for macrophage recruitment. In the absence of CD276 on tumor cells, significantly lower macrophage infiltration was observed (Fig. 3D). In a previous study conducted in an in vivo mouse model, reduced myeloid-derived suppressor cells in tumor was reported in CD276^*KO*^ mice^49^ that is consistent with our data. According to the phenotypic analysis of infiltrated macrophages in CD276^*KO*^ spheroids in comparison to CD276^*WT*^ spheroids, the reduction in infiltration is not associated with changes in macrophage polarization markers, although we cannot rule out possible effects on the metabolic level or other surface markers not-included in our analysis. Of note, as seen in Supplementary Fig. 4C, CD276^*KO*^ macrophages inside the CD276^*WT*^ spheroids display higher MFI of CD276 compared to their counterparts in CD276^*KO*^ spheroids, which could be due to tragocytosis as initially hypothesized. It is also noteworthy to mention, in our spheroid-macrophage coculture experiments, genetic editing of macrophages to knockout CD276 was not completely successful, and depending on the donor, up to 30% of macrophages still expressed detectable CD276. Despite of no correlation observed between the count of infiltrated macrophages versus percentage of CD276-expressing macrophages, we still cannot formally rule out a contribution of CD276^+^ macrophages to the spheroid recruitment.

When we screened for soluble proteins in the supernatant of these cultures, three major soluble factors were affected: Dkk-1, PAI-1 and uPA. The involvement of Dkk-1, negative regulator for Wnt signaling, in cell adhesion and migration has been implied but currently in debate regarding its role as PAI-1 modulator^50,51^. Therefore, we focused on uPA and PAI-1. Proteolytic activity of macrophage-produced uPA was demonstrated to be essential for matrix degradation and adhesion leading to macrophage infiltration in a 3D matrigel experimental setting^52^. However, in our 3D spheroid setting, addition of exogenous uPA neither restored the macrophage recruitment nor affected the PAI-1 concentration. Moreover, besides uPA, PAI-1 interacts with other proteins including the low-density lipoprotein receptor-related protein 1 (LRP1), and vitronectin. Indeed, the LRP1-PAI-1 interaction was proposed to be responsible for macrophage recruitment in an in vivo tumor model^54^, yet a recent publication challenged the previous observation by demonstrating that increased PAI-1 in skeletal muscle in aged mice resulted in delayed macrophage recruitment^55^. Despite using different models, these contradictory data indicate the necessity of further investigation to elaborate the involvement of PAI-1 in macrophage mobility. On the other hand, studies performed in cancer patients have been providing supportive data. A pan-cancer analysis of coagulome and TME environment across multiple tumor samples highlighted the positive correlation of CD276 to PAI-1 and uPA^56^. As in line with our data, a role of CD276^high^ TAM in triple-negative breast cancer patients was attributed to ECM modeling for immune cell infiltration^46^. However, whether PAI-1 acts as a checkpoint for macrophages to infiltrate in our setting requires further investigation.

We decided to further investigate CD276 biology by targeting it with specific antibodies. While using one of the antibodies currently tested in clinical trials would be more clinically relevant, accessing these antibodies are not easy. Hence, we used commercially available antibodies in our assays and this antibody might have different features than the ones in clinical trials. Targeting CD276 with specific antibodies in our spheroid-macrophage coculture resulted in increased macrophage numbers in spheroids, suggesting that CD276 favors in our setting for tumor-supportive macrophage. In this experiment, one striking result was the selective effect of αCD276 antibody treatment on CD276 expression on macrophages. In the presence of αCD276 antibody, its overall expression on macrophages was reduced. We ruled out the epitope competition between staining antibody and treatment antibody, since CD276 staining was not complete demolished, rather a reduction in expression was observed. Additionally, this shift in expression was only observed on macrophages but not on tumor cells in the same sample. As a result, we wanted to explore further the impact of targeting CD276 on macrophages. Since GM-CSF generated MDMs significantly expressed higher CD276, we included these cells as well in the further experiments.

The cytoplasmic tail of CD276 is short and has no known signaling motif, yet CD276 overexpression on tumor cells was reported to increase Jak2 and STAT3 phosphorylation^16^, therefore we speculated that any induction of downstream signaling might occur via a cis-partnership and one or more of the immunoreceptors expressed on macrophages are potential candidates. According to our phospho-immunoreceptor array experiment performed on macrophages, αCD276 treatment on M-CSF and GM-CSF MDMs induced different patterns of immunoreceptor phosphorylation. ILT2, ILT4 and ILT5 phosphorylation tend to be stronger in M-CSF MDMs, while LAIR-1, PECAM-1 and Siglec-3 phosphorylation was only detected in GM-CSF MDMs. All these receptors are inhibitory receptors, supporting the idea of CD276 being an inhibitory molecule. In both MDM subset varieties, the phosphorylation of FcγRIIα raised a question about Fc binding of the antibody. All of the experiments were conducted in Fcγ Receptor blocking conditions, yet still to clarify the unspecific involvement of Fcγ receptors, THP1^*WT*^ and THP1^*CD276KO*^ cells were treated with αCD276 antibody and FcγRIIα phosphorylation was only detected in THP1^*WT*^ cells indicating that crosslinking for this antibody is essential for its function (Supplementary Fig. 8). Our further analysis with kinase phosphorylation confirmed the differential impact of αCD276 on M-CSF and GM-CSF MDMs. Only in GM-CSF MDMs, αCD276 treatment induced CREB, HSP27, ERK1/2, RSK1/2/3, STAT3 and c-Jun phosphorylation. According to our tumor data, CD276 could be an upstream regulator for PAI-1. To understand whether this is universal or tumor specific, we stimulated macrophages under different conditions in the presence of αCD276 and quantified PAI-1 and uPA production. Overall, M-CSF generated MDMs produced more uPA than GM-CSF generated MDMs whereas the opposite was observed for PAI-1 production. In M-CSF generated MDMs, neither uPA nor PAI-1 was detectably affected upon αCD276 treatment in any condition. On the other hand, in GM-CSF MDMs, αCD276 treatment tended to increase the PAI-1 production that could reduce their migration ability in the tissue. The higher potency of αCD276 antibody treatment on GM-CSF MDMs in comparison to M-CSF MDMs could be due to a difference in expression of CD276 or the potential cis-partner.

**Figure 6:**
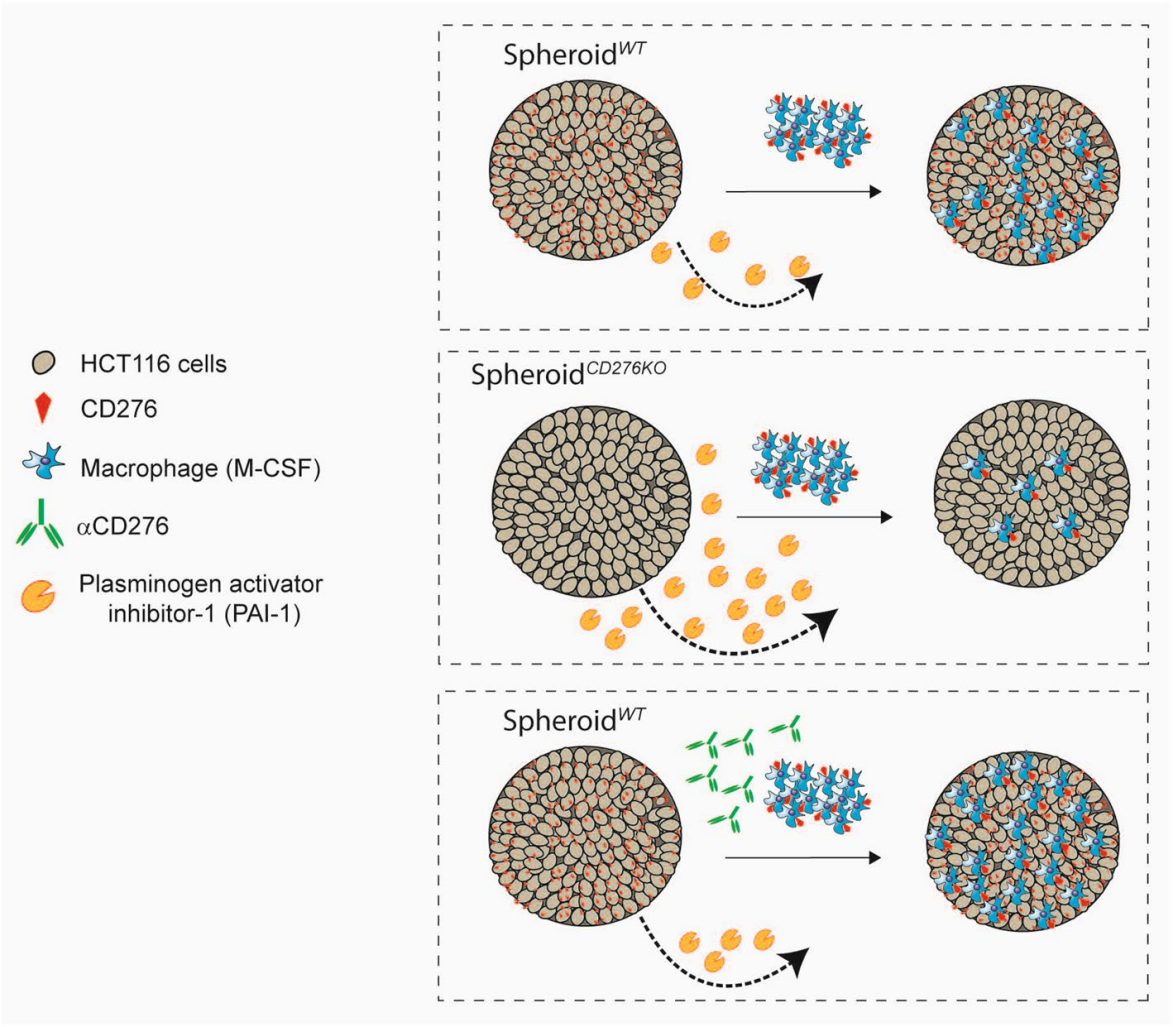
Graphical summary of the highlights. CD276 expression on tumor cells is an important player in macrophage recruitment and an upstream modulator for PAI-1 production in tumor microenvironment.

Overall, as summarized in Fig. 6, our study demonstrates a connection between CD276, macrophage recruitment and PAI-1 modulation in tumor, which is an important point to consider while developing αCD276-based immunotherapies. Additionally, our data suggest for the first time that a potential role of CD276 in initiating downsignaling in macrophages and we regard these data as a starting point to investigate further the CD276 function on macrophages.

## Supplementary Information

Supplementary Tables 1.

Supplementary Figures 2.
