# Supplementary material for: CD276 is an important player in macrophage recruitment into the tumor and an upstream regulator for PAI-1
Source: Sci Rep. 2021 Jul 21;11:14849. doi: 10.1038/s41598-021-94360-9 (PMC8295264; doi:10.1038/s41598-021-94360-9)
Supplement: Supplementary file 2 — Supplementary Figures 2. [file 41598_2021_94360_MOESM2_ESM.pdf]

Supp Fig 1

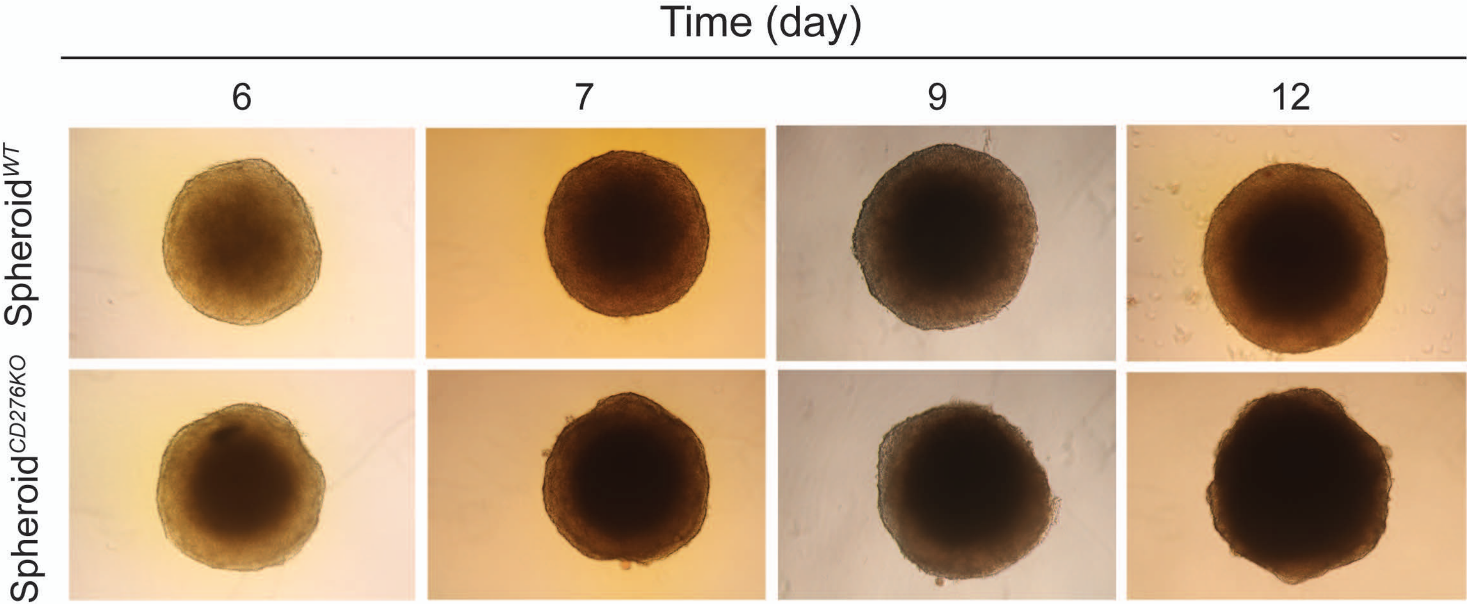

Supp Fig 2

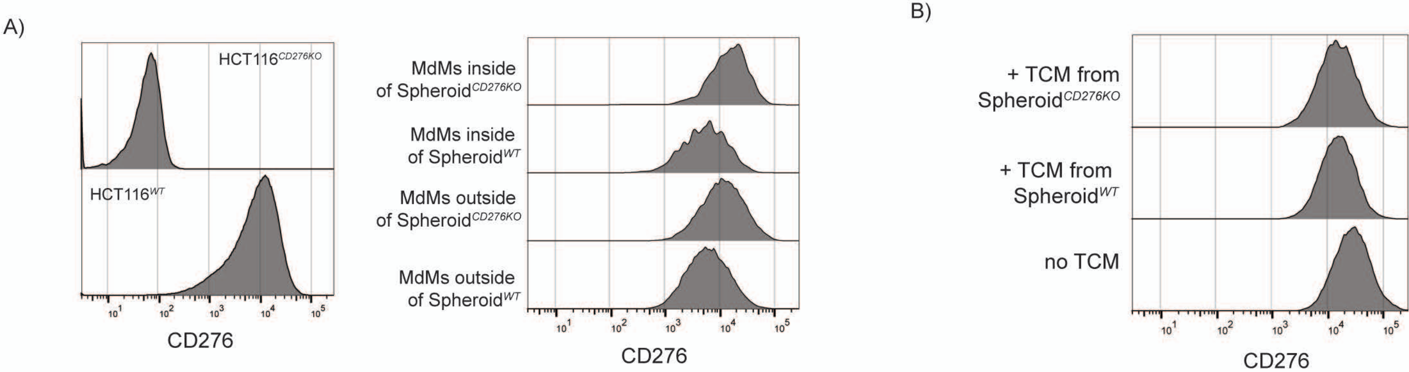
A)
HCT116^CD276KO
HCT116^WT
CD276
MdMs inside of Spheroid^CD276KO
MdMs inside of Spheroid^WT
MdMs outside of Spheroid^CD276KO
MdMs outside of Spheroid^WT
CD276
B)
+ TCM from Spheroid^CD276KO
+ TCM from Spheroid^WT
no TCM
CD276

Supp Fig 3

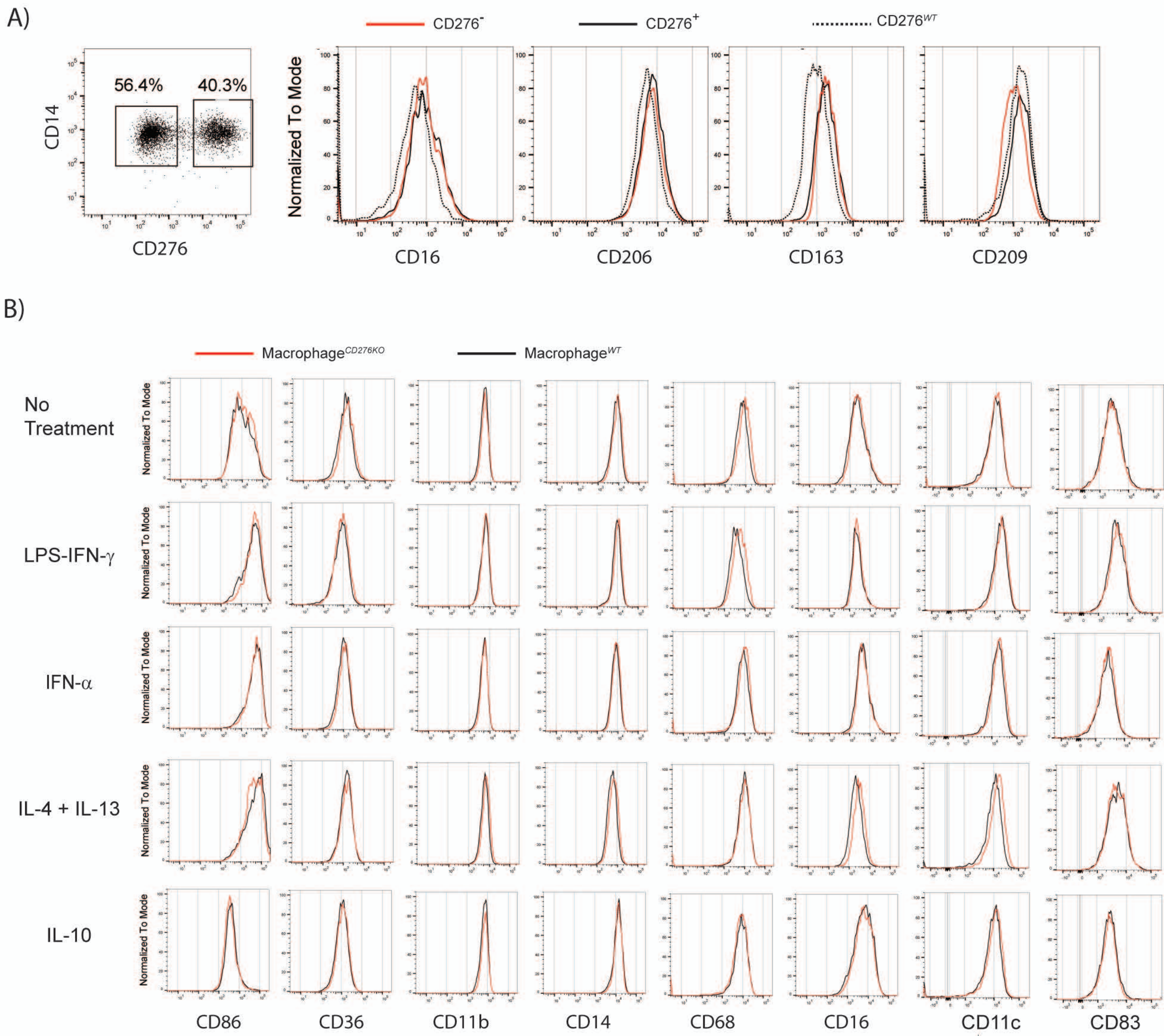

A)
CD276⁻
CD276⁺
CD276WT
56.4%
40.3%
CD14
CD276
Normalized To Mode
CD16
CD206
CD163
CD209
B)
Macrophage CD276KO
Macrophage WT
No Treatment
LPS-IFN-γ
IFN-α
IL-4 + IL-13
IL-10
Normalized To Mode
CD86
CD36
CD11b
CD14
CD68
CD16
CD11c
CD83

Supplementary Fig 3

C)

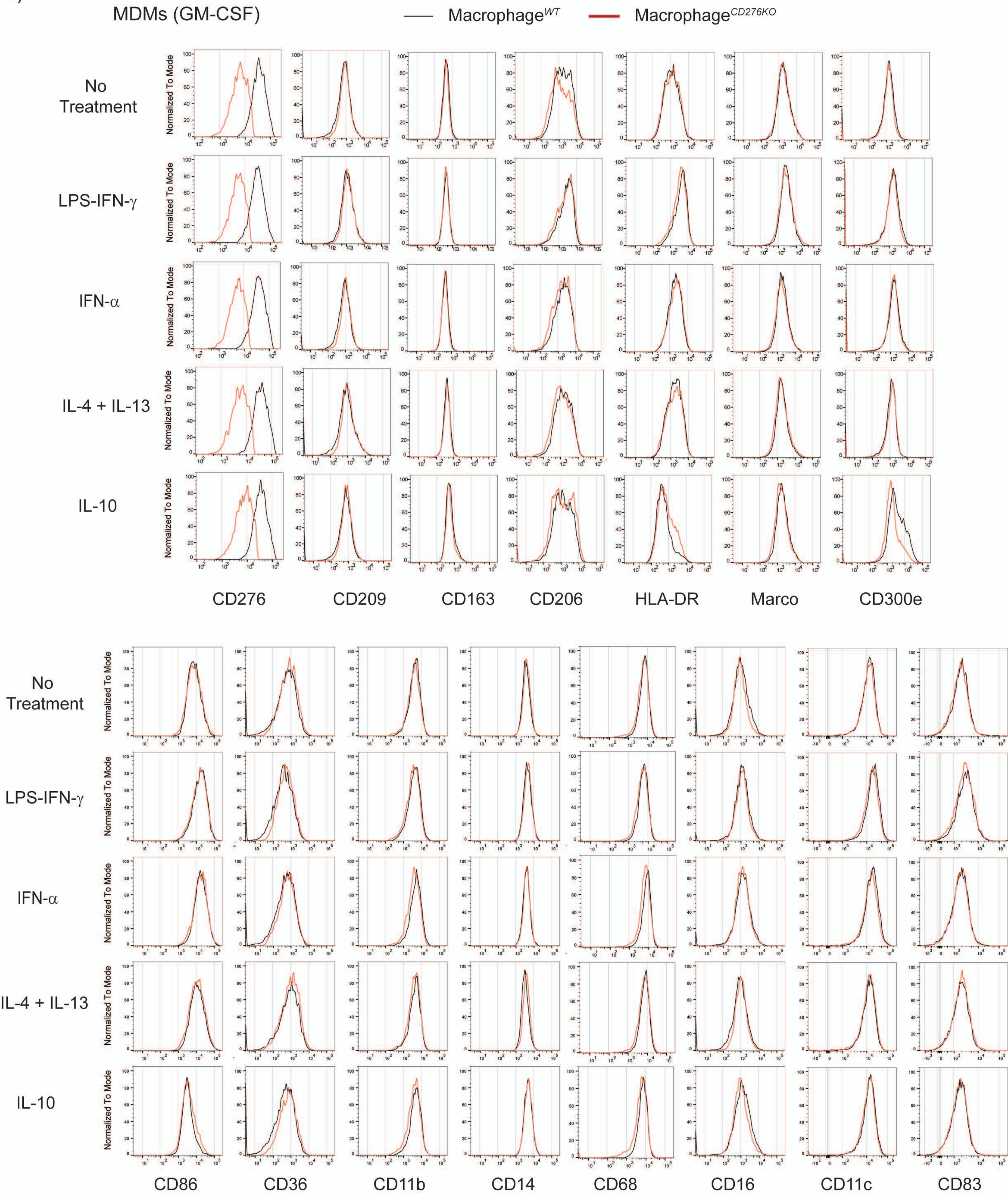

Supp Fig 4

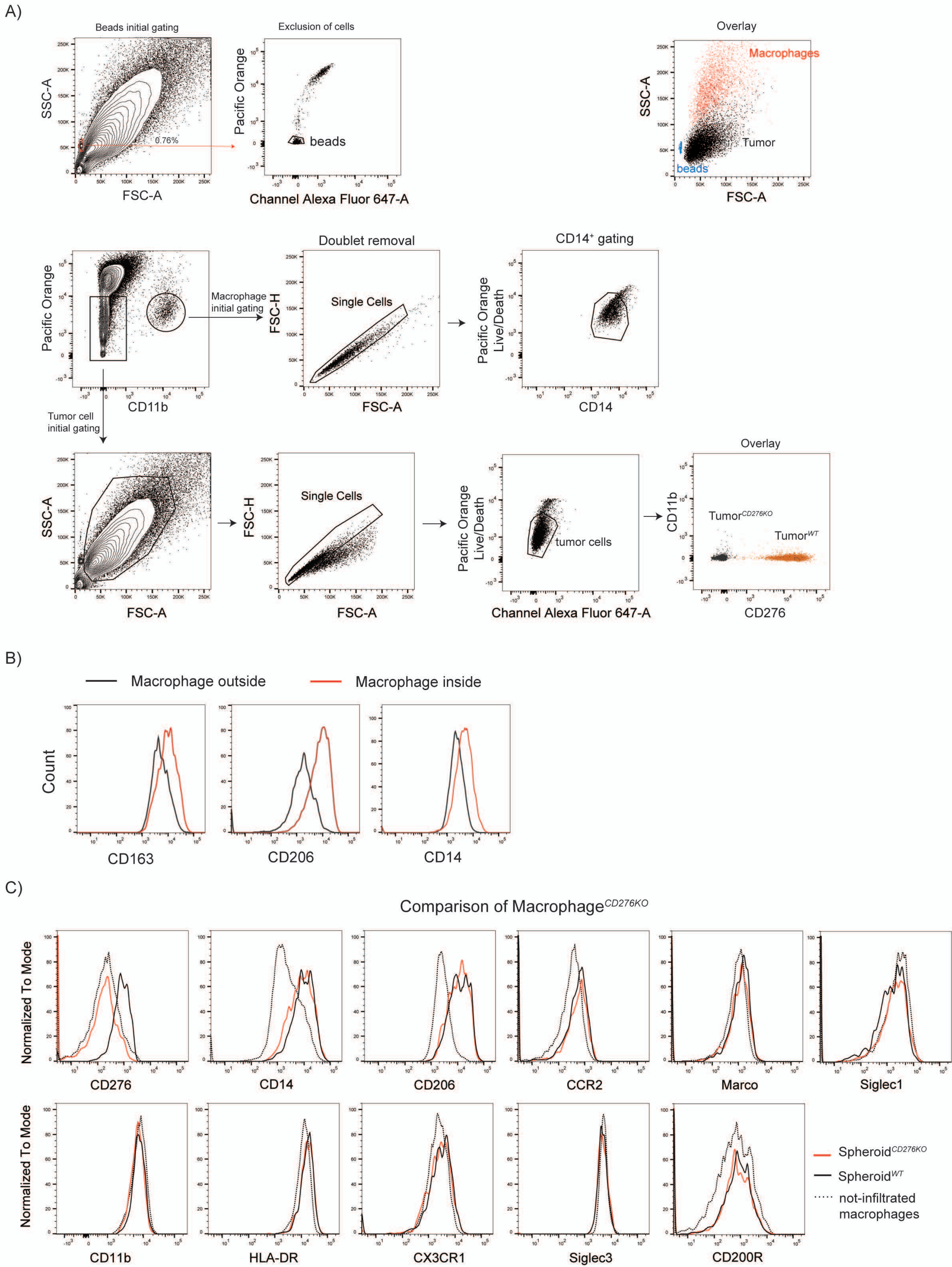
A)
Beads initial gating
SSC-A
FSC-A
0.76%
Exclusion of cells
Pacific Orange
beads
Channel Alexa Fluor 647-A
Overlay
Macrophages
Tumor
beads
SSC-A
FSC-A
Pacific Orange
CD11b
Macrophage initial gating
Tumor cell initial gating
Doublet removal
Single Cells
FSC-H
FSC-A
CD14+ gating
Pacific Orange Live/Death
CD14
SSC-A
FSC-A
Single Cells
FSC-H
FSC-A
Pacific Orange Live/Death
tumor cells
Channel Alexa Fluor 647-A
Overlay
CD11b
TumorCD276KO
TumorWT
CD276
B)
Macrophage outside
Macrophage inside
Count
CD163
CD206
CD14
C)
Comparison of MacrophageCD276KO
Normalized To Mode
CD276
CD14
CD206
CCR2
Marco
Siglec1
CD11b
HLA-DR
CX3CR1
Siglec3
CD200R
SpheroidCD276KO
SpheroidWT
not-infiltrated macrophages

Supp Fig 5

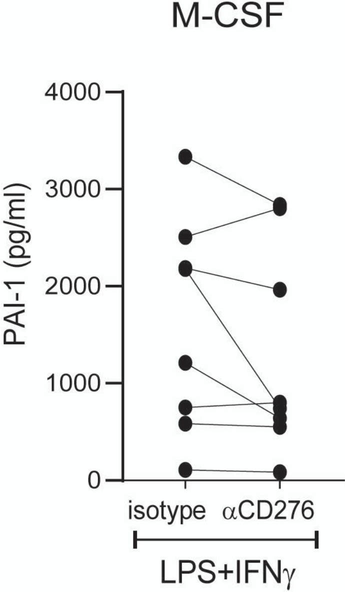

Supplementary Figure 6

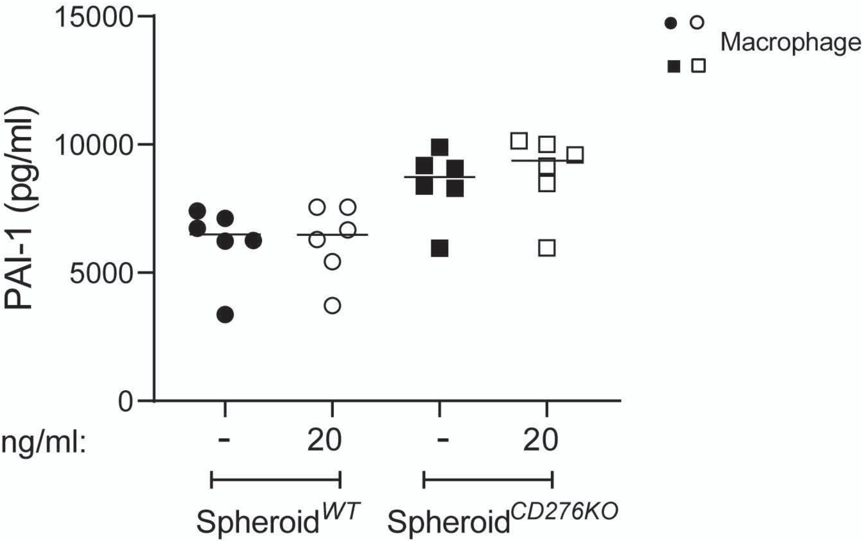

Supplementary figure 7

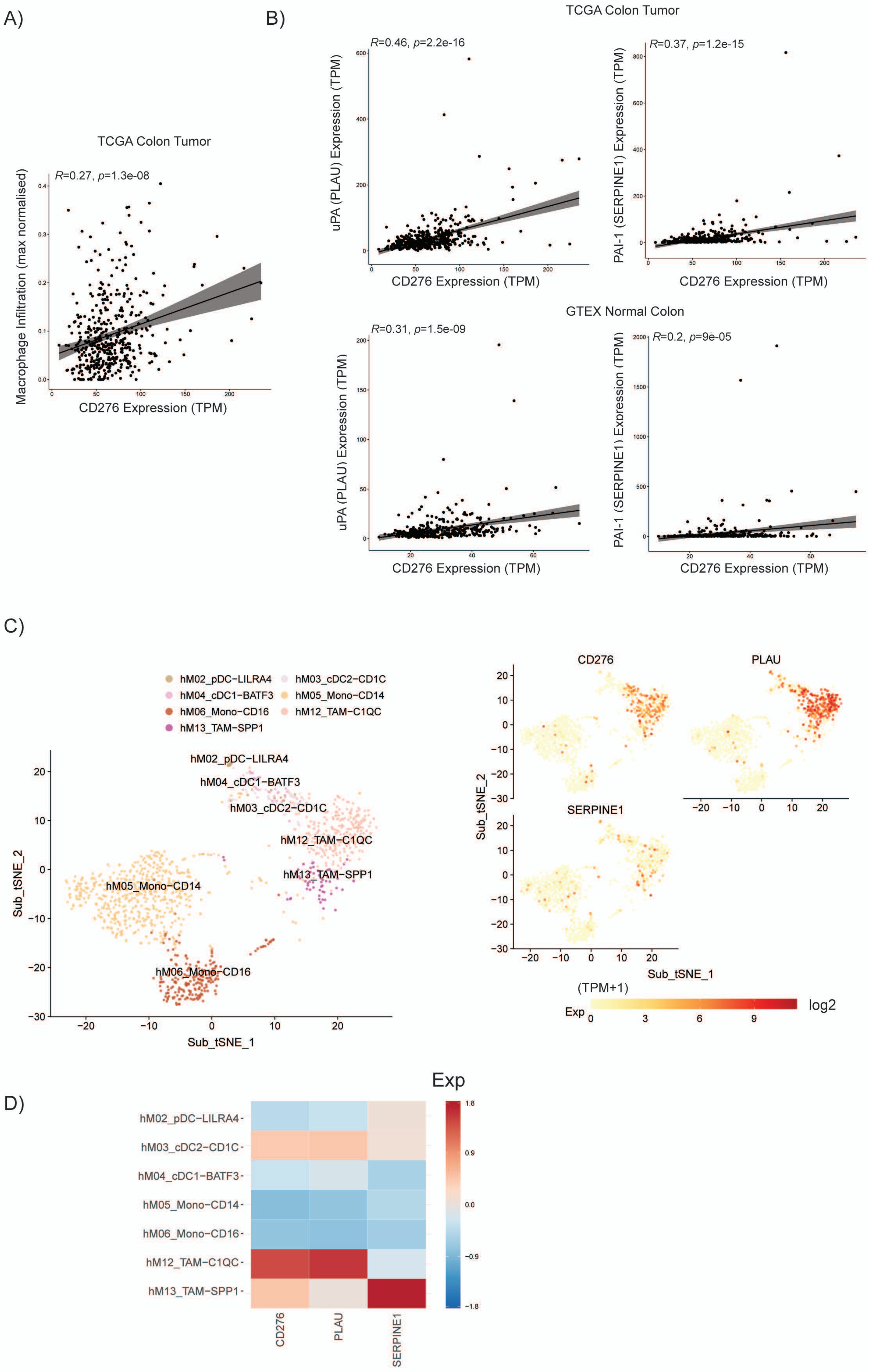

Supp Fig 8

A)

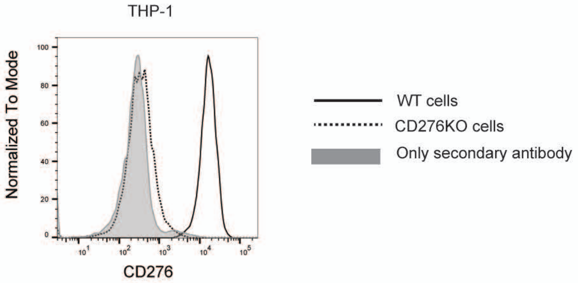
THP-1
Normalized To Mode
100
80
60
40
20
0
10^1
10^2
10^3
10^4
10^5
CD276
WT cells
CD276KO cells
Only secondary antibody

B)

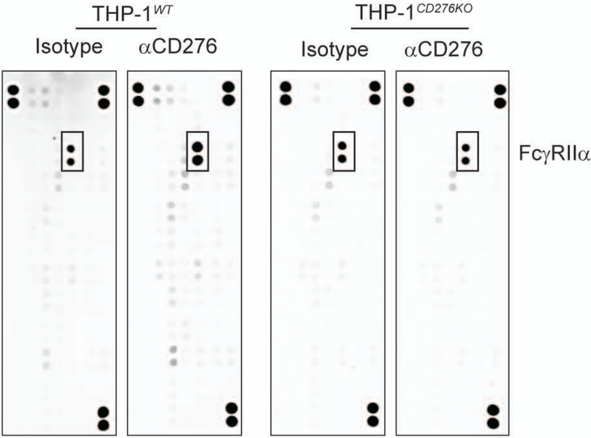
THP-1^WT
Isotype
αCD276
THP-1^CD276KO
Isotype
αCD276
FcγRIIα

Supplementary Fig 9

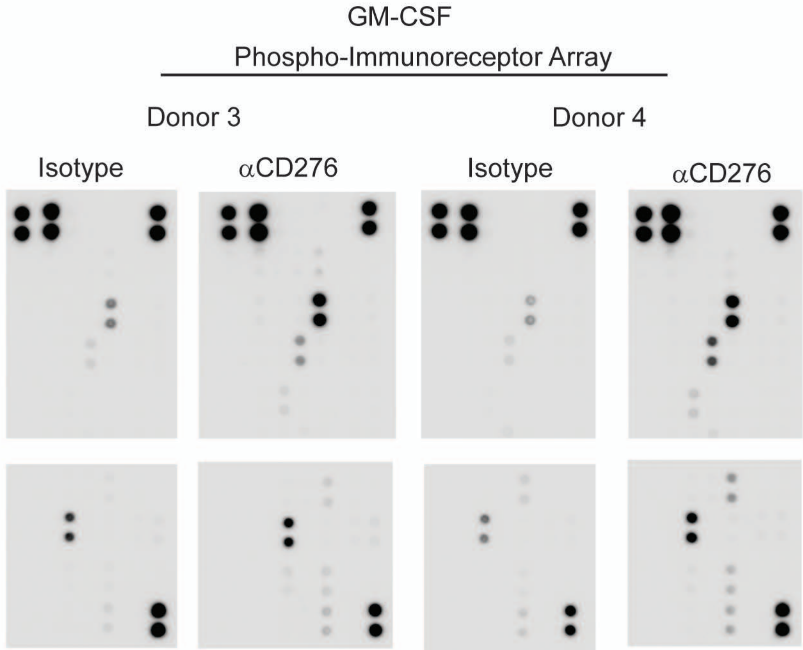

Supplementary Figures

Table S1: Crispr-Cas9 off-target *in silico* analysis: The CrisprOR software (http://crispor.tefor.net/)[34] was used to predict the off-target sides for CD276 specific gRNA (TTCGTGAGCATCCGGGATTTCGG). The table lists 66 potential off targets when up to four-mismatches are allowed. The program did not find any potential off-target with up to two-mismatches.

Supplementary Fig 1: Monitoring spheroids over time: HCT116^{WT} and HCT116^{CD276KO} cells were seeded and cultured for 5 days as described in materials and method. At day 5, medium was exchanged to serum-free medium and spheroids were cultured for additional 7 days, culturing in total 12 days. Pictures were taken with Canon Camera (EOS 1000D) attached to microscope with 5x objective magnification.

Supplementary Fig 2: CD276 upregulation on monocyte-derived macrophages exposed to tumor cells or tumor-conditioned medium (TCM). A) Freshly isolated monocytes were added to spheroids and cocultured. After 7 days coculture, spheroids were collected and monocyte-derived macrophages (MDMs) remaining in the wells were separately collected and pooled into one sample for each group. CD276 expression on tumor cells and MDMs inside and outside of the spheroids was analyzed by flow cytometry. **B)** Freshly isolated monocytes were seeded in ULA plates in 1:1 ratio for fresh medium:TCM. TCM was obtained from previously performed spheroid-monocyte cocultures, in which supernatant was collected at 7 days of coculture. The histograms in A and B represent one donor out of three MDM donors.

Supplementary Fig 3: Lack of CD276 expression does not affect the monocyte differentiation to macrophages or the further polarization of MDMs. A) Freshly isolated monocytes were genetically edited with Crispr-Cas9 technique either with scrambled gRNA as control (CD276^{WT}; dotted line) or with CD276-specific gRNA. After treatment, cells were cultured in GM-CSF supplemented medium and analyzed for the surface expression of CD276, CD14, CD16, CD163, CD206 and CD209. In the representative histograms, CD276^{-} (red line) and CD276^{+} (black line) fractions from genetically edited sample were compared to macrophages treated with control gRNA (CD276^{WT} dotted line). B&C) Freshly isolated monocytes were cultured for 7 days in M-CSF or GM-CSF supplemented medium. On day 7 cells were genetically edited either with scrambled gRNA as control (Macrophage^{WT}; black line) or CD276-specific gRNA (Macrophage^{CD276KO}; red line). 3 days after gene editing, MDMs were further treated with indicated stimuli for 24h and analyzed for indicated surface markers by flow cytometry. These histograms represent one representative donor out of three donors for B) M-CSF differentiated MDMs and C) GM-CSF differentiated MDMs.

Supplementary Fig 4: A) Gating strategy for infiltrated macrophages, beads and tumor cells. Pacific orange as live/dead staining agent was used to remove death cells. CD11b^{+} live cells and CD11b^{-} live cells were further analyzed to remove doublets. MDMs were further gated with CD14 expression. In parallel, tumor cells were further gated against pacific orange and an empty channel to avoid any dead cell contamination. Final plot indicates CD276 expression on tumor cells. **B)** Comparison of Infiltrated macrophages to macrophages outside of spheroids for CD163, CD206 and CD14. Red line: Macrophages inside, black line: macrophages outside. C) Histograms display the surface expression of indicated markers on Macrophage^{CD276KO} inside of Spheroid^{WT} (black line) versus Spheroid^{CD276KO} (red line) in comparison to not-infiltrated macrophages (CD276KO, dashed line) that were outside of the Spheroid^{WT}. The data represent one donor from 4 donors MDMs.

Supplementary Fig 5: PAI-1 production by MDMs (M-CSF). Monocytes were cultured in M-CSF supplemented medium for 7 days. After 7 days culture, MDMs were treated with LPS+IFNγ in the presence of αCD276 antibody or isotype control for 24h. The graph summarizes the concentration of PAI-1 in the supernatant. Each dot represents one donor.

Supplementary Fig 6: Addition of exogenous recombinant uPA did not affect the PAI-1 concentration in the spheroid-macrophage coculture. Spheroids formed from HCT116^{WT} or HCT116^{CD276KO} cells were cocultured with macrophages (7 days cultured in M-CSF supplemented medium, no gene editing) in the presence or absence of exogenous recombinant uPA protein (20 ng/ml; ab167714, Abcam) for 4 days. At the end of the experiment, supernatants were collected and analyzed for PAI-1 concentration with ELISA. The graphs summarize the concentration of PAI-1 from 6 different MDM donors, where each dot represent one donor.

Supplementary Figure 7: A) Correlation of CD276 expression with macrophage signature based on analysis of colon adenocarcinoma cohort in The Cancer Genome Atlas (TCGA). **B)** Coexpression analysis of CD276 and uPA (PLAU) or PAI-1 (SERPINE1) in colon adenocarcinoma cohort in TCGA and in normal tissue samples in GTEx platform. Pearson correlation and linear regression. C&D) Analysis of CD276, uPA (PLAU) and PAI-1 (SERPINE1) expression in myeloid cell subsets in previously published single-cell RNAseq data set generated with Smart-seq2 platform (available at http://crcleukocyte.cancer-pku.cn/). The graphs represent all the data generated from matching colon tumors, adjacent normal tissue and blood samples of the same patients. Clusters' names are obtained from the original publication. hM02-hM04: plasmacytoid DC (pDC), cDC2, and cDC1 cells. Blood-enriched monocyte subsets hM05-hM07: classical CD14^{hi}CD16^{-}, non-classical CD14^{+}CD16^{hi}, and intermediate CD14^{hi}CD16^{+} monocytes, respectively. Tumor-associated macrophages (TAM): hM12-hM13. C) t-SNE plot showing 7 myeloid clusters (left) and expression levels of CD276, uPA, and PAI-1 (right). D) Heatmap showing expression patterns of indicated genes for the 7 myeloid clusters.

Supplementary Fig 8: A) HCT116^{WT}, HCT116^{CD276KO}, THP-1^{WT} and THP1^{CD276KO} cells were stained with unconjugated primary rabbit anti-human CD276 (ab209895) followed by staining with secondary anti-rabbit antibody and analyzed by flow cytometry. **B)** THP-1^{WT} and THP-1^{CD276KO} cells were treated with αCD276 antibody for 10 min and cells lysates were prepared and processed for Phospho-Immunoreceptor array.

Supplementary Fig 9: The phosphor-immunoreceptor blots from Figure 5C with no correction on sharpness or contrast. After the contrast correction, the white circles around the dot blots in Figure 5C appears but this does not create or hide the detected signal.
